# Supplementary material for: Cdc42 mobility and membrane flows regulate fission yeast cell shape and survival
Source: bioRxiv. 2023 Jul 21:2023.07.21.550042. Preprint. [Version 1] doi: 10.1101/2023.07.21.550042 (PMC10370159; doi:10.1101/2023.07.21.550042)
Supplement: Supplement 5 [file NIHPP2023.07.21.550042v1-supplement-5.pdf]

bioRxiv preprint doi: https://doi.org/10.1101/2023.07.21.550042; this version posted July 21, 2023. The copyright holder for this preprint (which was not certified by peer review) is the author/funder, who has granted bioRxiv a license to display the preprint in perpetuity. It is made available under aCC-BY 4.0 International license.

# Supporting Information for

# Cdc42 mobility and membrane flows regulate fission yeast cell shape and survival

David M. Rutkowski[1], Vincent Vincenzetti[2], Dimitrios Vavylonis[1,]* and Sophie G. Martin[2, 3,]*

* Co-corresponding authors: Sophie G Martin, Dimitrios Vavylonis
**Email:** sophie.martin@unige.ch; vavylonis@lehigh.edu

**This PDF file includes:**

Figures S1 to S4
Tables S1 to S3
Legends for Movies S1 to S4
SI References

**Other supporting materials for this manuscript include the following:**

Movies S1 to S4

bioRxiv preprint doi: https://doi.org/10.1101/2023.07.21.550042; this version posted July 21, 2023. The copyright holder for this preprint (which was not certified by peer review) is the author/funder, who has granted bioRxiv a license to display the preprint in perpetuity. It is made available under aCC-BY 4.0 International license.

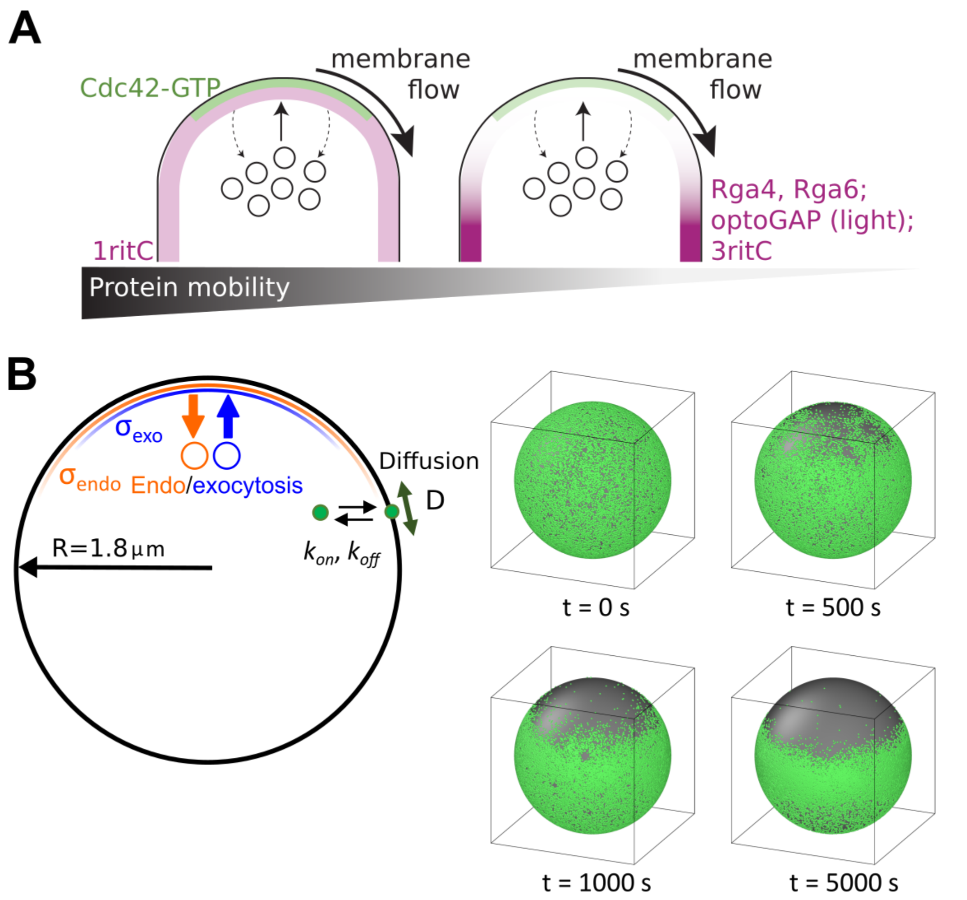


**Figure S1. In plane membrane flow due to localized secretion and broader endocytosis can lead to peripheral membrane protein depletion.**

(A) Schematics showing that high mobility proteins (Cdc42-GTP, 1ritC) are able to keep up with membrane flows due to exocytosis and endocytosis and remain at the tip of growing *S. pombe* cells. Low mobility proteins (Rga4/6, optoGAP which dimerizes under light, and 3ritC) are displaced by the membrane flow. (B) Computational model of membrane flow, reproduced from (1), simulated as stochastic, area-conserving exocytosis and endocytosis events centered at a point of spherical domain, with inert particles undergoing diffusion and binding/unbinding. Model shows depletion of initially uniform particle distribution away from the region where exocytosis and endocytosis are occurring, for sufficiently small diffusion coefficients and unbinding rates. The depletion phenomenon occurs when the exocytosis region is narrower than the endocytosis even when the overall rates of membrane secretion and internalization are equal to each other.

bioRxiv preprint doi: https://doi.org/10.1101/2023.07.21.550042; this version posted July 21, 2023. The copyright holder for this preprint (which was not certified by peer review) is the author/funder, who has granted bioRxiv a license to display the preprint in perpetuity. It is made available under aCC-BY 4.0 International license.

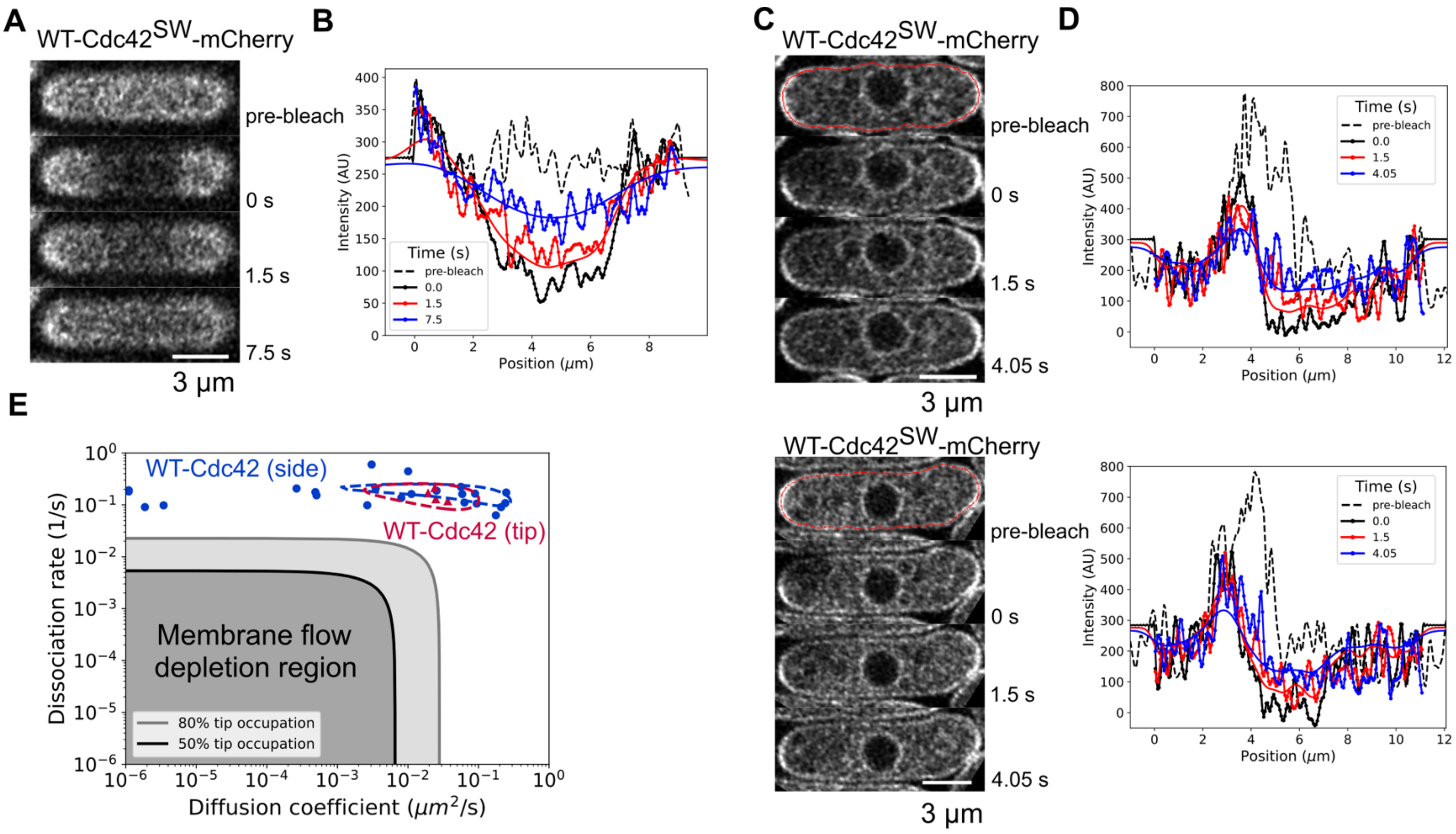


**Figure S2. FRAP fits to extract diffusion coefficients and membrane dissociation rates.**

(A) Photobleaching of a rectangular region along the cells sides. Confocal section at the cell surface for WT Cdc42-mCherry$^{SW}$. Similar FRAP experiments were performed for cells in Figure 2B. (B) Recovery traces over time and fit (solid lines) for cell in panel A. The fitted intensity was measured by projecting the intensity within a box of size the cell width perpendicularly to the long axis of the cell. (C) Two examples of half-tip bleach of WT Cdc42-mCherry$^{SW}$, imaged along a confocal section through the cell middle. (D) Recovery traces over time along with model fit (solid lines) for cells in panel C. The intensity was measured over a strip of width four pixels (1 pixel = 0.0516 μm) along a cell contour shown as red line in panel C. (E) Best fit parameters for membrane unbinding and diffusion for both side and tip recovery as in Figure 2B. Dashed line regions are drawn based on an average across all cell recoveries. Cdc42-GDP diffusion and dissociation constants are comparable to ~0.2 μm$^2$/s and ~0.03 /s measured using a different FRAP method in (5).

bioRxiv preprint doi: https://doi.org/10.1101/2023.07.21.550042; this version posted July 21, 2023. The copyright holder for this preprint (which was not certified by peer review) is the author/funder, who has granted bioRxiv a license to display the preprint in perpetuity. It is made available under aCC-BY 4.0 International license.

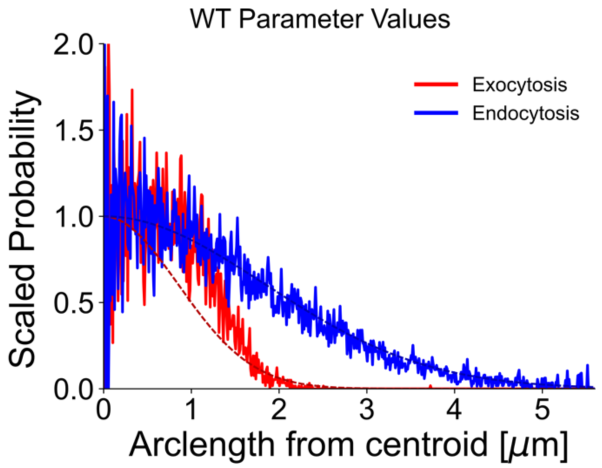


**Figure S3.** Steady state distribution of exocytosis and endocytosis in WT simulations of Figure 1E (solid lines) versus the desired distribution (dashed lines) in (1). Average was calculated similarly to concentration profiles over 5000 s at steady state.

bioRxiv preprint doi: https://doi.org/10.1101/2023.07.21.550042; this version posted July 21, 2023. The copyright holder for this preprint (which was not certified by peer review) is the author/funder, who has granted bioRxiv a license to display the preprint in perpetuity. It is made available under aCC-BY 4.0 International license.

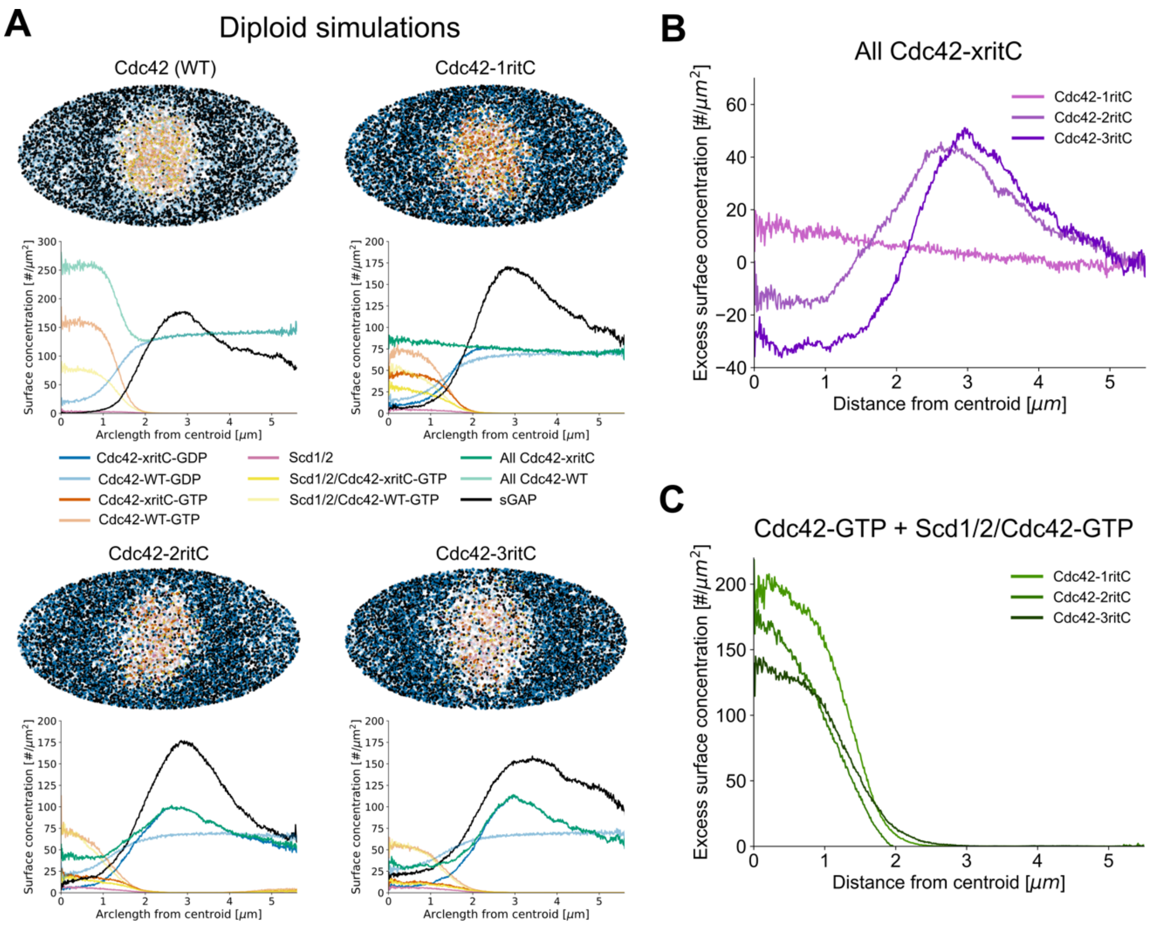


**Figure S4. Simulated polarization for diploid cell parameters.**

(A) Steady state snapshots and concentration profiles from diploid simulations after steady state for WT or where one copy (half of Cdc42) has lower mobility corresponding to either Cdc42-1ritC, Cdc42-2ritC, or Cdc42-3ritC. WT Cdc42 associated particles shown in faded colors. Profiles are averaged over time as in Figure 1. Snapshots were taken after 600 s. (B) Side normalized Cdc42 concentration of only the mutant Cdc42 component. (C) Side normalized Cdc42-GTP concentration for both mutant and WT components.

bioRxiv preprint doi: https://doi.org/10.1101/2023.07.21.550042; this version posted July 21, 2023. The copyright holder for this preprint (which was not certified by peer review) is the author/funder, who has granted bioRxiv a license to display the preprint in perpetuity. It is made available under aCC-BY 4.0 International license.

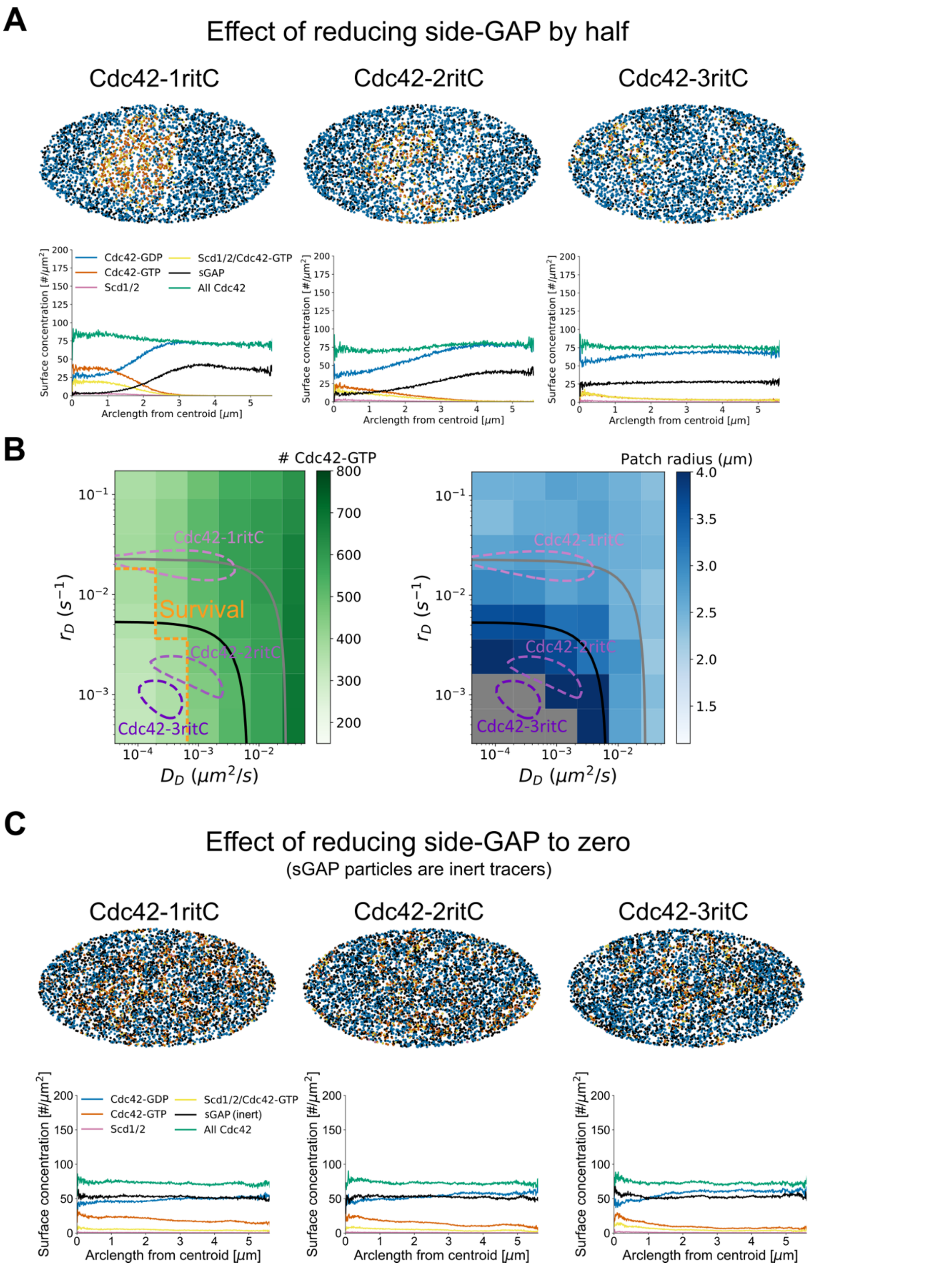


**Figure S5. Simulations with reduction of sGAP particle numbers.** (A) Steady state snapshots and concentration profiles for simulations where the number of sGAP has been reduced by ½ compared to Figure 1E. The simulation with Cdc42-ritC shows small transient Cdc42-GTP clusters that are picked up in the concentration profile. However, these small clusters are mobile over the simulation time and do not lead to a depletion of sGAP around them. Snapshots were

bioRxiv preprint doi: https://doi.org/10.1101/2023.07.21.550042; this version posted July 21, 2023. The copyright holder for this preprint (which was not certified by peer review) is the author/funder, who has granted bioRxiv a license to display the preprint in perpetuity. It is made available under aCC-BY 4.0 International license.

taken after 14000 s. (B) Parameter scan for amount of Cdc42-GTP and the patch width as in Figure 3E, for ½ sGAP simulations. The survival threshold line shrinks compared to Figure 3E. (C) Steady state snapshots and concentration profiles for simulations where the number of sGAP has been reduced to 0. In these simulations passive tracer sGAP particles were added to detect stable polarization. All cases show transient Cdc42-GTP clusters that are picked up in the concentration profile. These small clusters are mobile over the simulation time and do not lead to a depletion of inert tracer sGAP around them. Snapshots were taken at 15000 s.

bioRxiv preprint doi: https://doi.org/10.1101/2023.07.21.550042; this version posted July 21, 2023. The copyright holder for this preprint (which was not certified by peer review) is the author/funder, who has granted bioRxiv a license to display the preprint in perpetuity. It is made available under aCC-BY 4.0 International license.

## Table S1. Model parameter values for particle reactions.

| Variable | Value | Description |
|---|---|---|
| $\Delta t$ | 0.001 s | Timestep for simulations |
| $D_T$ | 0.025 μm$^2$/s | Diffusion coefficient of WT Cdc42-GTP (tip FRAP, this work) |
| $D_D$ | 0.1 μm$^2$/s | Diffusion coefficient of WT Cdc42-GDP (side FRAP, this work) |
| $D_S$ | 0.0025 μm$^2$/s | Diffusion coefficient of Scd1/Scd2 complex (WT cells) (same as Bem1 (2)) |
| $D_{ST}$ | 0.0025 μm$^2$/s | Diffusion coefficient of Scd1/Scd2/Cdc42-GTP complex (WT cells, same as Scd1/Scd2 complex) |
| $D_{sGAP}$ | 0.001 μm$^2$/s | Diffusion coefficient of sGAP (assumed to be inside membrane flow depletion region) |
| $r_T$ | 0.17 /s | Membrane dissociation rate of WT Cdc42-GTP (tip FRAP, this work) |
| $r_D$ | 0.17 /s | Membrane dissociation rate of WT Cdc42-GDP (tip FRAP, this work) |
| $r_S$ | 0.5 /s | Membrane dissociation rate of Scd1/2 (decreased from Bem1 value (2)) |
| $r_{ST}$ | 0.5 /s | Membrane dissociation rate of Scd1/2/Cdc42-GTP (decreased from (2)) |
| $r_{sGAP}$ | 0 /s | Membrane dissociation rate of sGAP (assumed to be inside membrane flow depletion region) |
| $k_{hydro}$ | 0.175 /s | Background hydrolysis rate (halved from (2) to account for sGAP contribution) |
| $k_D$ | $(r_T/2 + r_D)$ | Membrane binding rate of Cdc42-GDP (empirical estimate giving a significant fraction (~3/5) of all WT Cdc42 on plasma membrane at steady state) |
| $k_s$ | 0.01 /s | Membrane binding rate of Scd1/2 (decreased from (2) to match decrease in $r_S$) |
| $k_{ST}$ | 0.015 /s | Membrane binding rate of Scd1/2 in cytoplasm to membrane bound Cdc42-GTP (decreased from (2)) |
| ρ | 0.05 μm | Bimolecular interaction radius (2) |
| $\sigma$ | 0.055 μm | Bimolecular unbinding distance (close to ρ, (2)) |
| $\lambda_{SD}$ | 5.3 /s | Bimolecular rate for Scd1/2 + Cdc42-GDP on membrane (as for Bem1 complex (2)) |
| $\lambda_{ST}$ | 9.6 /s | Bimolecular rate for Scd1/2 + Cdc42-GTP on membrane (as for Bem1 complex (2)) |
| $\lambda_{STT}$ | 5 /s | Bimolecular rate for Scd1/2/Cdc42-GTP mediated activation of membrane bound Cdc42-GDP (lowered compared to (2)) |
| $\lambda_{sGAP}$ | 10 /s | Bimolecular rate parameter for sGAP mediated hydrolysis of Cdc42-GTP (large enough to matter) |
| $N_{Cdc42}$ | 5000 | Total number of Cdc42 particles (Estimates from total cell numbers in (3, 4) and (PomBase.org), divided by 4 for sphere volume and considering a larger fraction of Cdc42 is bound to internal membranes) |
| $N_{Scd1/2}$ | 225 | Total number of Scd1/2 particles (from Scd1 in (4) divided by 2, assumed to be tip biased) |
| $N_{sGAP}$ | 2250 | Total number of sGAP particles (essentially arbitrary since $\lambda_{sGAP}$ is tuned but comparable to sum of Rga4 = 646, and Rga6 = 1102 in (4)) |

bioRxiv preprint doi: https://doi.org/10.1101/2023.07.21.550042; this version posted July 21, 2023. The copyright holder for this preprint (which was not certified by peer review) is the author/funder, who has granted bioRxiv a license to display the preprint in perpetuity. It is made available under aCC-BY 4.0 International license.

**Table S2. Model parameter values for membrane flows from (1) unless otherwise indicated.**

| | | |
|---|---|---|
| $A_{exo}$ | 3.14 x $10^{-2}$ $\mu m^2$ | Surface area of individual exocytotic vesicle |
| $A_{endo}$ | 6.42 x $10^{-3}$ $\mu m^2$ | Surface area of individual endocytotic vesicle |
| $r_{exo}$ | 0.68 /s | Rate of exocytosis events per tip in growing *S. pombe* |
| $r_{endo}$ | 2.44 /s | Rate of endocytosis events per tip in growing *S. pombe* |
| $w_{exo}$ | 0.01 µm | Variance of blurring Gaussian for exocytosis (determined based on profiles from (5) and (1)) |
| $w_{endo}$ | 1.51 µm | Variance of blurring Gaussian for endocytosis (determined based on profiles from (5) and (1)) |
| $\alpha$ | 0.5 | Fraction of mobile component in membrane |
| $\gamma$ | 1.0 | Coupling between the proteins and the flowing membrane |
| $R_{cutoff}$ | 2.0 µm | Cutoff for effect of exo/endocytosis |

bioRxiv preprint doi: https://doi.org/10.1101/2023.07.21.550042; this version posted July 21, 2023. The copyright holder for this preprint (which was not certified by peer review) is the author/funder, who has granted bioRxiv a license to display the preprint in perpetuity. It is made available under aCC-BY 4.0 International license.

**Table S3. Strain list**

| Strain number | Genotype | Reference |
|---|---|---|
| YSM3138 | h+ leu1-32 cdc42-mCherry$^{SW}$:Term:kanMX ura4-294:pshk1:CRIB-3GFP:ura4+ | (5) |
| YSM2468 | h+ leu1-32 cdc42-mCherry$^{SW}$-1ritC:kanMX ura4-294:pshk1:CRIB-3GFP:ura4+ | (5) |
| YVV4074 | h+/h- ura4-/ura4+ ade6-M210/ade6-M216 cdc42-mCherry$^{SW}$-3ritC:Term:kanMX/cdc42+ | This work |
| YSM4075 | h+/h- ura4-/ura4+ ade6-M210/ade6-M216 cdc42-mCherry$^{SW}$-2ritC:Term:kanMX/cdc42+ | This work |
| YSM4077 | h+/h- ura4-D18 ade6-M210/ade6-M216 leu1-32:pshk1:CRIB-3GFP:ura4+:leu1+/leu1+ cdc42-mCherry$^{SW}$-3ritC:Term:kanMX/cdc42+ | This work |
| YSM4078 | h+/h- ura4-D18 ade6-M210/ade6-M216 leu1-32:pshk1:CRIB-3GFP:ura4+:leu1+/leu1+ cdc42-mCherry$^{SW}$-2ritC:Term:kanMX/cdc42+ | This work |
| YSM4081 | h+/h- ura4- ade6-M210/ade6-M216 leu1-32:pshk1:CRIB-3GFP:ura4+:leu1+/leu1- rga6Δ::bleMX/rga6+ rga4Δ::natMX/rga4+ rga3Δ::hphMX/rga3+ cdc42-mCherry$^{SW}$-3ritC:kanMX | This work |
| YSM4083 | h+/h- ura4-D18 ade6-M210/ade6-M216 leu1-32/leu1-32:pshk1:CRIB-3GFP:ura4+:leu1+ cdc42-mCherry$^{SW}$-1ritC:kanMX/cdc42+ | This work |
| YSM4084 | ura4? ade6- cdc42-mCherry$^{SW}$-2ritC:Term:kanMX | This work |
| YSM4085 | ura4-D18 ade6- leu1-32:pshk1:CRIB-3GFP:ura4+:leu1+ cdc42-mCherry$^{SW}$-2ritC:Term:kanMX | This work |
| YSM4087 | ade6- rga6Δ::bleMX rga4Δ::natMX rga3Δ::hphMX cdc42-mCherry$^{SW}$-3ritC:kanMX leu1-32:pshk1:CRIB-3GFP:ura4+:leu1+ | This work |

bioRxiv preprint doi: https://doi.org/10.1101/2023.07.21.550042; this version posted July 21, 2023. The copyright holder for this preprint (which was not certified by peer review) is the author/funder, who has granted bioRxiv a license to display the preprint in perpetuity. It is made available under aCC-BY 4.0 International license.

**Movie S1.** Simulation with WT cell parameters (same as Figure 1E) from 0 to 500 s. 2D Mollweide projection of particles on a sphere.

**Movie S2.** Simulation with Cdc42-1ritC haploid cell parameters (same as Figure 3A) from 0 to 1500 s. 2D Mollweide projection of particles on a sphere.

**Movie S3.** Simulation with Cdc42-2ritC haploid cell parameters (same as Figure 3A) from 0 to 5000 s. 2D Mollweide projection of particles on a sphere.

**Movie S4.** Simulation with Cdc42-3ritC haploid cell parameters (same as Figure 3A) from 0 to 9000 s. 2D Mollweide projection of particles on a sphere.

## SI References


1. V. Gerganova *et al.*, Cell patterning by secretion-induced plasma membrane flows. *Sci Adv* **7**, eabg6718 (2021).
2. M. Pablo, S. A. Ramirez, T. C. Elston, Particle-based simulations of polarity establishment reveal stochastic promotion of Turing pattern formation. *PLoS Comput Biol* **14**, e1006016 (2018).
3. S. Marguerat *et al.*, Quantitative analysis of fission yeast transcriptomes and proteomes in proliferating and quiescent cells. *Cell* **151**, 671-683 (2012).
4. A. Carpy *et al.*, Absolute proteome and phosphoproteome dynamics during the cell cycle of Schizosaccharomyces pombe (Fission Yeast). *Mol Cell Proteomics* **13**, 1925-1936 (2014).
5. F. O. Bendezu *et al.*, Spontaneous Cdc42 polarization independent of GDI-mediated extraction and actin-based trafficking. *PLoS Biol* **13**, e1002097 (2015).
